# Relationship between immediate postpartum umbilical cord pH, fetal distress and neonatal outcome

**DOI:** 10.12669/pjms.36.7.2536

**Published:** 2020

**Authors:** Wajeeha Syed, Nazia Liaqat, Qudsia Qazi, Sumaira Yasmeen

**Affiliations:** 1 Dr. Wajeeha Syed, FCPS, Assistant Professor, Gynae Unit, Department of Gynae, Medical Teaching Institute, Lady Reading Hospital, Peshawar, KPK, Pakistan; 2 Dr. Nazia Liaqat, FCPS, Assistant Professor, Gynae Unit, Department of Gynae, Medical Teaching Institute, Lady Reading Hospital, Peshawar, KPK, Pakistan; 3 Dr. Qudsia Qazi, FCPS, Assistant Professor, Gynae Unit, Department of Gynae, Medical Teaching Institute, Lady Reading Hospital, Peshawar, KPK, Pakistan; 4 Dr. Sumaira Yasmeen, Assistant Professor, Gynae Unit, Department of Gynae, Medical Teaching Institute, Lady Reading Hospital, Peshawar, KPK, Pakistan

**Keywords:** Fetal Distress, Hypoxia, Acidemia, Umbilical cord pH, Neonatal outcome

## Abstract

**Objectives:**

To determine relationship between immediate postpartum umbilical cord pH, fetal distress and neonatal outcome

**Methods:**

This descriptive cross-sectional study was conducted in the department of Gynaecology, Lady Reading Hospital Peshawar, Pakistan, from January 2019 to July 2019. This study included 27 full-term pregnant women who had abnormal CTG during the active or latent phase of labour. Data were analyzed by IBM SPSS Statistics for Windows, Version 23.0.

**Results:**

Out of 27, most patients 13 (48.14%) were in the age group 20-25 years,11 (40.74%) to 26-30 years and 3 (11.11%) belonged to 31-35 years of age group. CTG abnormalities were severe bradycardia, late deccelerations and persistent variable deccelerations with loss of baseline variability. Of all delivered babies, 21 (77%) babies had birth weight<3.5 kg and 6 (22%) had >3.5 kg birth weight. 20 (74.07%) had acidosis (pH <7.2) at the time of birth, of which one had severe hypoxemia and acidosis with pH 6.85. APGAR score at 0 minutes showed a strong positive correlation (r=0.818, p= <0.001) with cord pH, while APGAR at five minutes was also strongly correlated (r= 773, p=<0.001). Of all babies 18(66.66%) with PH less than 7.2 were admitted in NICU while only 2 babies with PH more than 7.2 were admitted. (p value= 0.005).

**Conclusion:**

Low umbilical cord pH values of babies born by cesarean section (for fetal distress) are strongly correlated with low APGAR score at birth and higher rates of NICU admission.

## INTRODUCTION

Birth asphyxia is one of the top three causes of neonatal morbidity and mortality in structurally normal term babies. The other two causes are neonatal sepsis and respiratory distress syndrome.^1^ Perinatal asphyxia can be the cause of hypoxic-ischemic encephalopathy, cerebral palsy, seizure disorder and developmentally delayed child.^2^ APGAR scoring practice has been ordinarily formulated to quickly summarize the condition of newborn against infant mortality.^3^ Shortly after birth the APGAR score is done at 1 and 5 minutes of age to evaluate the newborn status. This is widely used and is universally accepted technique Yet, this practice is not appropriate for the evaluation of birth asphyxia because it could be affected by various other factors like prematurity, maternal sedation, or analgesia, muscle disease, cerebral malformations and cardiorespiratory conditions, where without any birth asphyxia score may be low.^4^

Umbilical cord blood gas analysis is a really important tool, and it is believed to be the most excellent predictor of birth asphyxia soon after birth.^5^ In accordance with the American College of Obstetricians and Gynaecologists committee point of view on umbilical cord blood gas analysis, sampling of cord blood for acid base status is recommended when an intrapartum event might be accompanied with deleterious consequences.^6^ Cord blood gas analysis is a vital criterion for determining a causal connection between intrapartum asphyxia and the consequent occurrence of cerebral palsy. To differentiate between asphyxiated and normal new born lactate and pH value are the best parameters.^7^

An important association exist between umbilical cord pH, low APGAR score and incidence of selective neonatal outcomes such as neonatal intensive care unit admission and the need for advance resuscitation.^8^ Reducing fetal distress, fetal morbidity and mortality is a golden aim in obstetrics. The widespread use of intrapartum electronic fetal monitoring in this regard has contributed to increased cesarean section rate for presumed fetal distress. Fetal distress is the main indication for category one cesarean sections, which have been known to be associated with low APGAR score compared to non-category 1 cesarean sections.^9^,^10^

This study was conducted in a public sector tertiary care hospital with limited resources. It was conducted with the intention that positive outcomes in terms of improved neonatal survival will help in funding and designing protocols for measuring umbilical cord pH in babies with fetal distress. So that instead of sending all babies with presumed fetal distress to nurseries, referring neonates with low pH only, will reduce the workload on already overburdened NICUs.

## METHODS

This was a descriptive cross-sectional study conducted in the department of Obstetrics and Gynaecology, Lady Reading Hospital Peshawar, Pakistan, from January 2019 to July 2019. Twenty-seven pregnant women with singleton term pregnancy were included in the study. Informed consents were taken from included cases. Sampling was non probability convenient sampling. All the included women having signs of fetal distress like (fetal bradycardia, tachycardia, decelerations, meconium-stained liquor on artificial or spontaneous rupture of membranes) during active or latent phase of labour and for whom caesarean was decided as mode of delivery were considered as study cases. Women with intrauterine fetal demise, anomalous babies and with multiple gestation were excluded from the study.

After birth of baby by cesarean section, a portion of the umbilical cord was double clamped, and one ml of blood was drawn from the umbilical artery in a pre-heparinized syringe which was sent to laboratory right away for testing umbilical cord arterial pH. After cord blood sampling, pH analysis was done within 20 minutes. All neonates born by cesarean section were attended by a senior resident of the neonatology unit of the hospital. APGAR score of all the newborn was evaluated at one and five minutes after birth. APGAR scores below seven was labeled as low APGAR score. The incidence and etiologies of neonatal morbidities were recorded. These findings were then recorded on pre-designed proforma along with age and gravidity of mother, birth weight of baby and admission to the NICU.

Babies with acidemia or low APGAR score or both were referred to NICU for further review by senior neonatologists. Acidemia was defined as cord blood pH less than 7.2. The study protocol was approved by the Ethical Committee of Lady Reading Hospital, Peshawar (Ref: No. 61, dated 15-02-2019). Data was analyzed using IBM SPSS Statistics for Windows, Version 23.0 (IBM Corp., Armonk, NY). Continuous variables were reported as mean and standard deviation and categorical variables as number (percentages). Chi-square test and Pearson’s correlation test were applied. The level of significance was set at P < 0.05. Sample size was calculated using confidence interval of 95%, Margin of error 10% and proportion of cesarean sections for fetal distress as 8%.^11^

## RESULTS

Out of all patients 13 (48.14%) women belonged to 20-25 years age group, 11 (40.74%) to 26-30 years and 3 (11.11%) belonged to 31-35 years of age group. (Table-I) Of all included women, 17 (62.69%) were primigravida. Gravida 2-4 were 3 in numbers each, (11.11%) and only 1(3.70%) was gravida 7. (Table-II)

**Table-I:** Age distribution.

Age groups	Numbers	Percentages
20-25 yrs	13	48.14%
26-30yrs	11	40.74%
31-35 yrs	03	11.11%

**Table-II:** Gravidity of patients.

Gravidity	Numbers	Percentages
Primigravida	17	62.69%
Gravida 2	03	11.11%
Gravida 3	03	11.11%
Gravida 4	03	11.11%
Gravida 7	01	3.7%

Patterns of CTG abnormalities leading to caesarean sections were, severe fetal bradycardia (FHR <100 bpm) in 03(11.11%), Late deccelerations in 22 (81.48%) and persistent variable decelerations with loss of baseline variability in 02 (7.41%) cases. Of all delivered babies, 21 (77%) babies had a birth weight<3.5 kg, and 6 (22%) had >3.5 kg birth weight. Of all babies, 20 (74.07%) had acidosis (pH<7.2) at the time of birth, of which one had severe hypoxemia and acidosis with pH 6.85.

**Fig.1:**
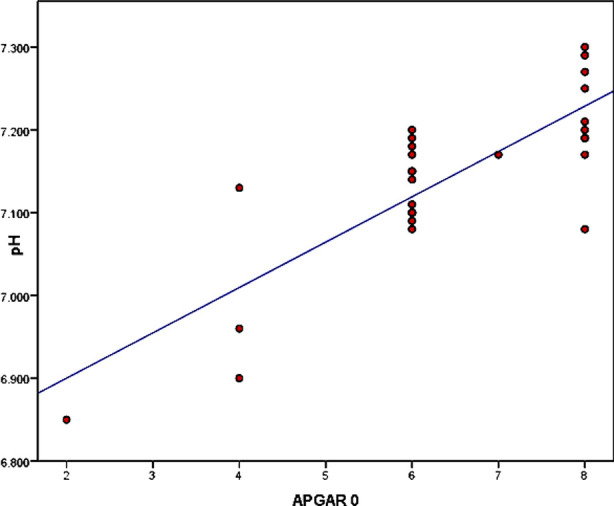
Correlation of APGAR 0 with pH.

By correlating APGAR score at 0 minutes with Umbilical cord pH it showed a strong positive correlation (r=0.818, p= <0.001) while APGAR at 5 min was also strongly correlated (r= 773, p=<0.001) shown in Fig. 1, 2 respectively Table-IV.

**Fig.2:**
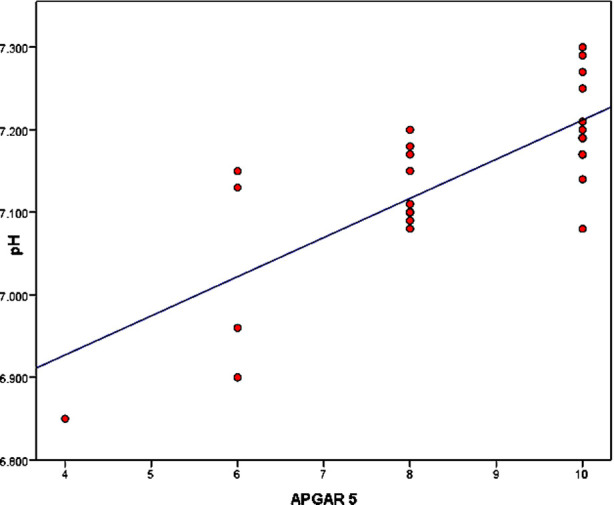
Correlation of Apgar 5 with pH.

**Table-III:** Patterns of CTG abnormalities.

Type of CTG abnormality	Number	Percentages
Severe bradycardia	03	11.11%
Late deccelerations	22	81.48%
Persistent variable deccelerations with loss of beat to beat variability	02	07.41

**Table-IV:** Outcomes in relation to birth weight.

Birth	Mean	Mean	Mean	NICU

weight	APGAR 0	APGAR 5	PH	Admission
<3.5kg	6.56±1.15	8.87±1.25	7.15±0.93	13(48.14%)
>3.5kg	6.18±2.0	8.0±2.19	7.12±0.12	7(25.92%)

Twenty babies got admitted to NICU, their mean APGAR score at 0 minute was 5.85±1.46, while APGAR at 5 min was 8±1.71. The majority of babies, 18(66.66%) with pH less than 7.2 were admitted in NICU while only 2 babies with pH more than 7.2 were admitted. (p value= 0.005) Table-V.

**Table-V:** Correlation of NICU admission with umbilical cord pH.

NICU admission			

PH	Yes	No	P-value
<7.2	18(66.66%)	2(7.40%)	0.005
>7.2	2(7.40%)	5(18.51%)	

## DISCUSSION

In this study neonatal acidemia at birth was present in 20(74.07%) cases. Out of these 20 babies having acidosis at birth, 18 got admitted to NICU due to birth asphyxia. It was noted that there was a statistically significant association between low APGAR and neonatal acidemia, indicating that these two variables of neonatal outcomes complement each other in the assessment of neonatal morbidity. Also there was a noteworthy association between 1st and 5th minute APGAR score, the umbilical cord artery pH and NICU admissions, as 20 babies admitted to NICU had APGAR score less than 7 at 0 minutes. Also this study showed correlation between abnormal CTG patterns and neonatal acidemia. Similarly, a correlation between cord pH less than 7.25 and neonatal outcome has been found in another study done in Pakistan.^12^ However the study done by Naina Kumar et al.^13^, showed that adverse neonatal outcomes are not associated with non-reassuring fetal heart rate or MSL. According to Younas and colleagues, severity of metabolic acidosis with pH less than 7.01 is strongly associated with serious neonatal neurological morbidity and neonatal mortality.^14^ Locatelli et al.^15^ estimated the predictive value of umbilical artery acidemia in term infants and found that acidemia is present in 38% of term babies with low APGAR score and it is chiefly related with intrauterine vascular disease such as pre-eclampsia, placental abruption, or birth weight and placental vascular pathologies. Lower umbilical cord blood pH is associated with unfavorable immediate outcome in terms of neurological abnormalities and poor final outcomes in terms of death at the time of discharge. Similar short-term outcome association of APGAR score and cord blood pH was reported by other authors.^16^-^18^ Association between intrapartum fetal distress, cord acidemia and neonatal hypoglycemia in babies admitted to NICU has been found out by Durrani and colleagues.^19^

Apgar score for neonatal assessment at birth is a universal practice while cord blood gas analysis is reserved for high risk situations or where there is low APGAR score at birth. Sabol and colleagues found increased rates of neonatal acidemia with obstetric events like presence of meconium, placental abruption and cesarean deliveries despite having normal 5-minute Apgar scores. Additionally, fetal acidemia with pH less than 7.0 was associated with increased risk of respiratory distress syndrome, NICU admissions.^20^

### Limitations of the study

It was a small single center study conducted on small number of babies with fetal distress delivered by cesarean section. Larger prospective multicenter studies are needed to confirm the causal relationship between umbilical cord acidemia and neonatal outcomes.

## CONCLUSION

Cord blood pH is the most sensitive parameter for diagnosis of birth asphyxia and should be performed in all high-risk births, as this may help in providing appropriate care to the newborn at birth and in preventing as well as decreasing neonatal morbidity and mortality.

### Authors’ Contribution:

**WS:** Conceived the idea, collected data and manuscript writing and is responsible for the accuracy and integrity of work.

**NL:** Collected data and edited manuscript. **QQ & SY:** Reviewed and finalized manuscript.
